# Supplementary material for: Immunological Profile and Markers of Endothelial Dysfunction in Elderly Patients with Cognitive Impairments
Source: Int J Mol Sci. 2024 Feb 4;25(3):1888. doi: 10.3390/ijms25031888 (PMC10855959; doi:10.3390/ijms25031888)
Supplement: Supplementary file 1 [file ijms-25-01888-s001.zip › ijms-2791794-supplementary.pdf]

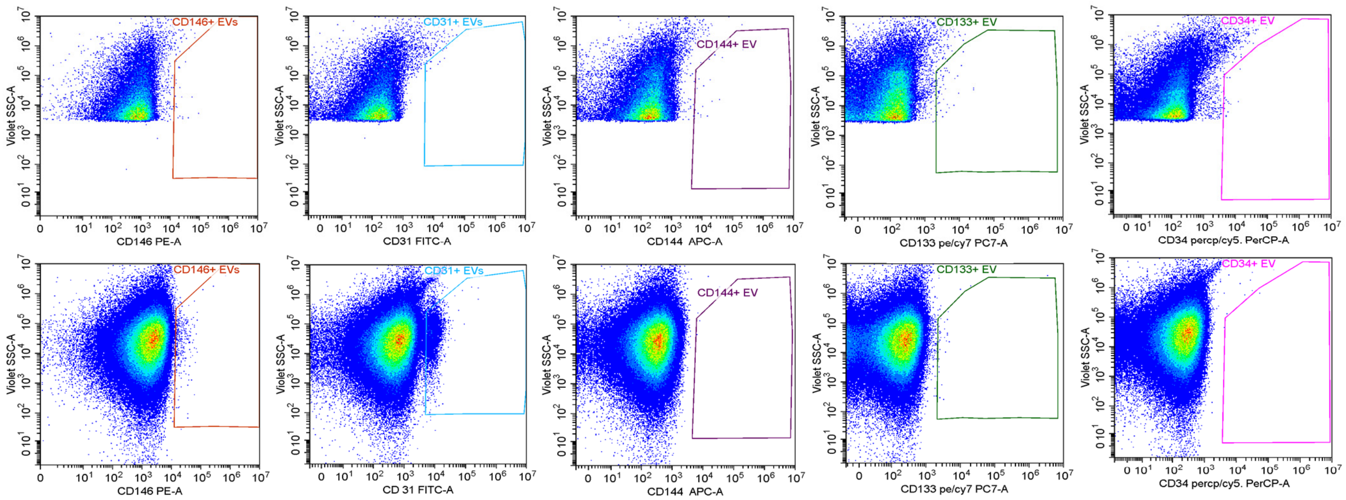

Supplementary Figure S1. Representative pseudo color dot plots of the EVs phenotyping results (bottom row) compared to appropriate fluorescent monoclonal antibodies (upper row). Columns representing appropriate EVs: 1. CD 146+ EV. 2. CD31+ EV. 3. CD144+EV. 4. CD133 EV. 5. CD34 EV.
